# Fatal Cytomegalovirus Disease after Combination Therapy with Corticosteroids and Rituximab for Granulomatosis with Polyangiitis

**DOI:** 10.1155/2015/538137

**Published:** 2015-01-29

**Authors:** Talal Hilal

**Affiliations:** Department of Internal Medicine, University of Kentucky College of Medicine, Charles T. Wethington Building 304B, 900 South Limestone Street, Lexington, KY 40536, USA

## Abstract

The association of cytomegalovirus (CMV) with autoimmune disease is poorly understood with suggested causality and reported viral reactivation coinciding with active inflammation. We report a case of a patient who presented with diffuse alveolar hemorrhage and acute renal failure from rapidly progressive glomerulonephritis ultimately diagnosed with granulomatosis with polyangiitis (GPA). She was acutely managed with plasmapheresis to reduce antibody-mediated end-organ damage, hemodialysis for worsening hyperkalemia and acidosis, and high-dose intravenous methylprednisolone. She was transitioned to oral prednisone and started on weekly rituximab with resultant remission induction over a three-week period at which point she developed reactivation of CMV causing severe fatal lung disease and viremia. The case highlights the multiple factors associated with CMV reactivation in cases of severe systemic inflammatory states and the need for further research to help establish practice guidelines regarding antimicrobial prophylaxis in patients with autoimmune diseases on prolonged courses of corticosteroids and biologic agents.

## 1. Introduction

Granulomatosis with polyangiitis (GPA) is a small-vessel vasculitis characterized by necrotizing granulomatous inflammation in the upper and/or lower respiratory tract and glomeruli. Circulating anti-neutrophil cytoplasmic antibodies (ANCAs) predominantly directed against the neutrophil serine proteinase- (PR-) 3, but also myeloperoxidase (MPO), is seen in 80–94% of affected patients [1]. The cause of this disease and other autoimmune conditions remains unclear with several postulated theories. An association with cytomegalovirus (CMV) has been reported as an etiologic trigger. Furthermore, CMV reactivation is being diagnosed more frequently in active autoimmune disease with resulting deleterious effects. We report a case of a patient who presented with manifestations of severe pulmonary-renal syndrome (PRS) secondary to GPA and who developed fatal CMV disease causing severe pneumonitis and viremia three weeks into her hospital stay and discuss the factors that contribute to CMV reactivation and the potential indications for antimicrobial prophylaxis.

## 2. Case Report

A 55-year-old female presented to a local emergency department with a 2-week history of progressively worsening shortness of breath and 1 episode of hemoptysis on day of admission. Her past medical history was significant for severe chronic sinusitis for which she took multiple courses of antibiotics over the preceding four-month period. She denied smoking, alcohol, or illicit drug use. Vital signs revealed tachycardia and tachypnea, with a temperature of 36.7°C, heart rate of 120/min, blood pressure of 130/75 mmHg, respiratory rate of 26 breaths/min, and oxygen saturation of 92% on ambient air. Physical examination revealed an obese female in mild respiratory distress. Examination of the chest revealed bilateral inspiratory and expiratory crackles. Cardiovascular exam revealed normal heart sounds without murmurs and moderate to severe lower extremity edema extending to the thighs without overlying chronic skin changes. Abdominal exam was unremarkable.

**Figure 1:**
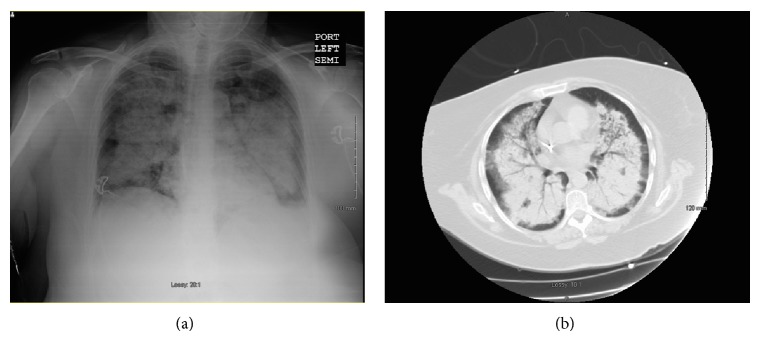
(a) Chest X-ray showing bilateral pulmonary infiltrates in an airspace pattern. (b) Computed tomography of the chest without contrast showing airspace opacities bilaterally sparing the periphery.

Initial laboratory evaluation revealed a leukocyte count of 16 k/*μ*L, hemoglobin of 6.8 g/dL, platelet count of 318 k/*μ*L, creatinine of 5.2 mg/dL, blood urea nitrogen of 68 mg/dL, and a potassium of 7 mmol/L. Chest X-ray revealed diffuse ground glass opacities bilaterally (Figure 1(a)). Computed tomography (CT) of the chest without contrast revealed diffuse airspace disease bilaterally across upper and lower lung fields sparing the periphery (Figure 1(b)). The patient's presentation of acute respiratory failure and kidney injury was worrisome for autoimmune PRS. The differential diagnosis included GPA, microscopic polyangiitis (MPA), and Goodpasture's syndrome. Her history of chronic sinusitis made GPA the most likely diagnosis. Autoimmune work-up, including ANCAs, PR3, MPO, anti-glomerular basement membrane antibody, and antinuclear antibody, was requested.

The patient was started on pulse-dose steroids with 1 gram of intravenous methylprednisolone daily for 3 days followed by 1 mg/kg of oral prednisone and emergently received renal replacement therapy in the form of hemodialysis for worsening metabolic acidosis and hyperkalemia. She was then transferred to our institution where she was intubated and placed on mechanical ventilation upon arrival for worsening respiratory failure. She was started on plasmapheresis with fresh frozen plasma (FFP) replacement to decrease the level of circulating immunoglobulins mediating neutrophil activation and vascular inflammation. Diagnostic bronchoscopy revealed DAH. Autoimmune work-up revealed positive c-ANCA, and the PR3 level was 1351 (normal 0–19 AU/mL) confirming the diagnosis of GPA. Treatment with intravenous rituximab was initiated in addition to steroids and plasmapheresis. Based on anecdotal experience, the patient was started on trimethoprim-sulfamethoxazole (TMP-SMX) and fluconazole for prophylaxis against* Pneumocystis jirovecii* and fungal disease, respectively.

**Figure 2:**
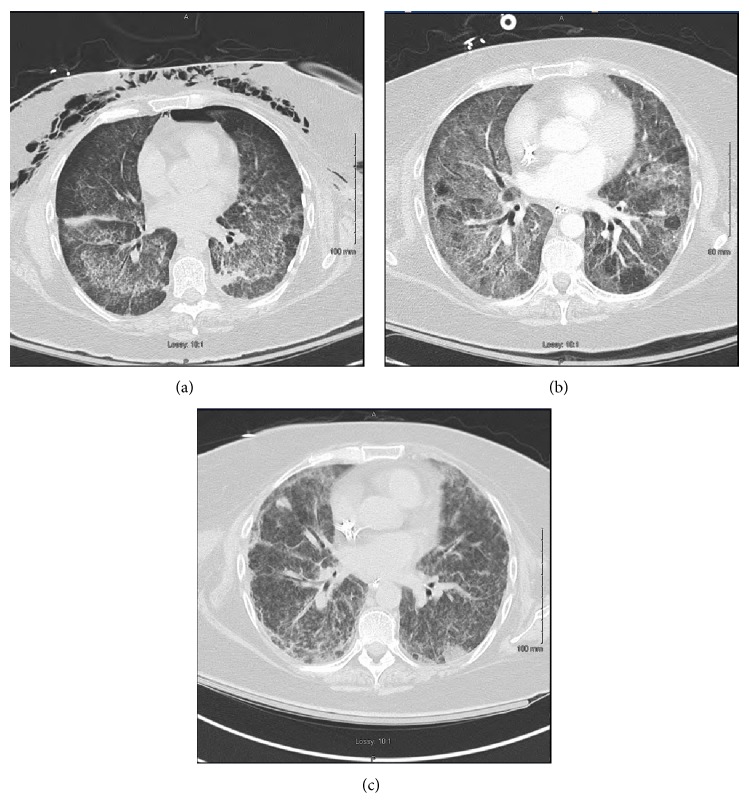
(a) CT of the chest without contrast on Day 10 showing marked improvement in airspace opacities, (b) Day 30 showing increase in diffuse ground glass opacities with associated reticular abnormality, (c) and Day 40 showing further increase in ground glass opacities with evidence of interstitial fibrosis.

On Day 10 of hospital stay, CT of the chest revealed marked improvement in airspace disease (Figure 2(a)). The patient at that point was extubated. She had received three days of IV methylprednisolone with transition to oral prednisone, five treatments of plasmapheresis, and two doses of rituximab. On Day 20 of hospital stay, she had received two additional plasmapheresis treatments and a third dose of rituximab. Her PR3 level had decreased from 1351 on admission to 767 on Day 10 and 383 on Day 20. The patient was requiring intermittent hemodialysis but was recovering lung function.

On Day 25 of hospital stay, the patient developed acute respiratory decompensation and required reintubation and mechanical ventilation. It was thought that she had developed fluid overload and was in need of further ultrafiltration. However, the patient's gas exchange did not show improvement after three days of hemodialysis with a net negative fluid balance. She received her fourth and final dose of rituximab afterwards and was weaned off the ventilator and extubated on Day 30. To rule out the possibility of a pulmonary embolus, a repeat CT of the chest with contrast was obtained and revealed worsening airspace and interstitial lung disease bilaterally (Figure 2(b)).

Following extubation, the patient was having high oxygen requirements and complaining of severe shortness of breath on minimal exertion. A repeat PR3 level was lower at 310 on Day 30, indicating that symptoms were unlikely to be related directly to active GPA. A repeat diagnostic bronchoscopy with bronchial washings and lung biopsy was positive for CMV on BAL and tissue culture. Polymerase chain reaction (PCR) testing in plasma for quantification of CMV DNA was positive with a value of 188,000 International Units/mL. The patient was initiated on ganciclovir and IV CMV immunoglobulin for CMV pneumonitis and viremia. Her respiratory status continued to deteriorate and she required reintubation a week after starting antiviral therapy.

On Day 40, her PR3 level had decreased further to 150, but repeat CT scan of the chest without contrast revealed worsening airspace and interstitial disease (Figure 2(c)). At that point, the family decided to withdraw aggressive measures in favor of comfort care. Antivirals, steroids, and dialysis were stopped and the patient expired within a week. An autopsy was denied.

## 3. Discussion

Cytomegalovirus (CMV) is a ubiquitous virus belonging to the Herpesviridae family, which includes herpes simplex viruses (HSV) types 1 and 2, varicella zoster virus (VZV), and Epstein-Barr virus (EBV) [2]. The prevalence of CMV seropositivity in the United States increases with age from 36.3% in 6–11-year-olds to 90.8% in those aged >80 years [3]. The virus is typically acquired early in life from contact with bodily fluids and manifests in the two main forms of disease—primary infection, which is usually subclinical in immunocompetent hosts, and endogenous infection in CMV-seropositive individuals who experience reactivation from latency [2].

Endogenous infection from reactivation is always a concern with immunosuppression as seen in patients with hematologic malignancies or solid tumors who are receiving immunosuppressive chemotherapy and particularly transplant recipients. As a result, guidelines were developed for antimicrobial prophylaxis in neutropenic patients and transplant recipients. The two approaches in preventing CMV disease in those particular patient populations who are CMV seropositive are universal prophylaxis and preemptive therapy. With the prophylactic approach, patients receive antiviral therapy for a defined period of time, while, with the preemptive approach, CMV DNA monitoring is carried out every 1 to 2 weeks and patients are treated when viral DNA is detected or is on the rise on repeat testing [4–7].

The association of CMV with autoimmune disease has been described in two nonmutually exclusive ways: as a causal relationship wherein CMV triggers the pathogenesis autoimmune disease and as a complication of autoimmune disease wherein active inflammation causes endogenous CMV infection from reactivation. The evidence for the latter comes from immunocompetent patients with critical illness admitted to the intensive care unit and highlights the systemic inflammatory state as a possible trigger [8]. Approximately a third of these patients with latent infection develop reactivation of CMV [9]. This has been linked to impaired natural killer (NK) cell activity [10] and high levels of circulating tumor necrosis factor- (TNF-) *α*-producing T helper 1 cells [11, 12]. Along the same thought process, the septic stimuli that cause a systemic inflammatory response are also as seen in autoimmune diseases, acute graft rejection, and graft versus host disease (GVHD), all of which have a higher incidence of CMV reactivation.

In cases of ANCA-associated vasculitides, such as GPA, CMV infection seems to be related to the onset of inflammatory disease or to the initiation of immunosuppressive therapy [13–16], as seen in the present case. The distinction between the two is a difficult one to make. In examining the present case, the immunosuppressive effect was probably related to prednisone use rather than rituximab. Initial pulsed dosing of methylprednisolone was unlikely to have contributed as described in a study by Chibane et al. [17], which examined the tolerance profile associated with the three-day use of high-dose intravenous pulse methylprednisolone. A total of 146 patients were followed prospectively for 6 months. The majority (88.4%) developed complications, mostly neuropsychiatric (e.g., insomnia), but also cardiac (e.g., hypertension, bradycardia), and metabolic (e.g., hyperglycemia, hyperkalemia). There were no reports of infections even with such high doses. The immunosuppressive effect of corticosteroids seems to be related to the duration and not the quantity. The present patient was on high doses of oral prednisone for three weeks prior to onset of new symptoms.

Rituximab, the anti-CD20 monoclonal antibody, is becoming more widely utilized as a treatment option for GPA. Studies examining CMV reactivation rates in patients receiving rituximab are conflicting. Patel et al. [18] examined the incidence of CMV in 55 patients who received rituximab in the postrenal transplant setting and failed to show a difference between rituximab- and non-rituximab-treated patients. The rituximab-treated group did, however, have an increased incidence of other viral and fungal infections. Aksoy et al. [19] examined 64 case reports of patients with B-cell non-Hodgkin's lymphoma who developed serious viral infections after receiving rituximab and found that CMV was the second most common encountered infection, after hepatitis B. Case reports seem to be confounded by multiple associated risk factors that would predispose patients to CMV reactivation in addition to rituximab, including hematologic malignancies and transplantation [20, 21].

The exact mechanism of CMV reactivation in the present case points towards a multifactorial process. The use of prednisone very likely played a role, but the duration of treatment prior to reactivation is less than what would be expected in cases of reactivation in the posttransplant setting. Rituximab-induced CMV reactivation is a possibility with the aforementioned growing body of evidence in support of herpes viral reactivation, but the temporal relation and onset of viral illness remain unclear. Although the disease was under control when symptoms of CMV disease appeared, as evident by a stable PR3 level, the initial severe inflammatory state the patient presented with strongly supports it as an etiologic factor that mediated initial reactivation of CMV disease (see Table 1). Other factors that must not be overlooked include the underlying viral load [9] and the location of inflammation (i.e., lungs) [22].

Antimicrobial prophylaxis that includes TMP-SMX, fluconazole, and acyclovir/ganciclovir is currently used in patients with critical illness due to autoimmune disease based on anecdotal evidence, as seen in the present case. This highlights the need for research that seeks to determine the incidence of these opportunistic infections in patients with severe manifestations of autoimmune disease and those on prolonged corticosteroid therapy and biologic agents. Ultimately, structured practice guidelines need to be instituted to aid the physician in deciding whether to use antimicrobial prophylaxis in a somewhat overlooked patient population.

## 4. Conclusion

Endogenous infection from CMV reactivation is mediated by several factors in the setting of severe illness and inflammatory states. Guidelines currently do not exist for antimicrobial prophylaxis in patients with autoimmune diseases receiving prolonged courses of corticosteroids or biologic agents. The need to revisit the issue is essential, and larger studies need to be undertaken to examine the incidence of CMV disease in this particular population. The use of antiviral prophylaxis or establishing a preemptive therapy approach may prove to be an effective measure that prevents morbidity and mortality.

## Figures and Tables

**Table 1:** Time course of illness with corresponding PR3 level, treatment regimen, and reactivation of CMV disease.

	Day 5	Day 10	Day 20	Day 25	Day 30	Day 40
PR3 level	1351	767	383		310	150

Corticosteroid	Prednisone 80 mg	Prednisone 80 mg	Prednisone 80 mg	Prednisone 70 mg	Prednisone 70 mg	Prednisone 70 mg

Rituximab		Second dose	Third dose		Fourth dose	

Symptoms and signs of CMV disease				Hypoxia, fever	Worsening pulmonary infiltrates	Persistent viremia
